# Insect Odorant-Binding Proteins (OBPs) and Chemosensory Proteins (CSPs): Mechanisms and Research Perspectives in Mediating Insecticide Resistance

**DOI:** 10.3390/biology14101452

**Published:** 2025-10-20

**Authors:** Qian Wang, Xuping Shentu, Xiaoping Yu, Yipeng Liu

**Affiliations:** Zhejiang Provincial Key Laboratory of Biometrology and Inspection and Quarantine, College of Life Science, China Jiliang University, Hangzhou 310018, China; 19975264309@163.com (Q.W.); stxp@cjlu.edu.cn (X.S.); yxp@cjlu.edu.cn (X.Y.)

**Keywords:** odorant-binding proteins, chemosensory proteins, insecticide, resistance mechanism, insecticide resistance

## Abstract

**Simple Summary:**

The development of insecticide resistance in insects poses a significant threat to global food production. While previous research has primarily focused on mechanisms such as target-site mutations and enhanced metabolic detoxification, this review emphasizes a novel perspective: certain proteins traditionally associated with insect olfaction and taste also contribute to insecticide resistance. These proteins can sequester insecticide molecules, assist in detoxification processes, and even mediate behavioral avoidance of treated plants. Understanding this emerging role opens promising opportunities. By targeting these specific proteins, scientists can develop more precise and environmentally sustainable pest control strategies, such as designing novel chemicals that disrupt their function or employing gene-silencing technologies. This work holds significant value as it provides innovative approaches to combat resistant pests, safeguard crop yields, and support the advancement of sustainable agriculture.

**Abstract:**

Insecticide resistance has become a critical issue threatening global agricultural production and food security. Previous studies have primarily focused on resistance mechanisms such as target-site mutations, enhanced metabolic detoxification, and reduced cuticular penetration. However, growing evidence in recent years indicates that odorant-binding proteins (OBPs) and chemosensory proteins (CSPs)—beyond their roles in chemoreception—also play key roles in the development of insecticide resistance. Research has revealed that these proteins significantly modulate insect susceptibility to insecticides through various mechanisms, including direct binding to insecticides, regulation of detoxification metabolic pathways, and influence on behavioral adaptations in pests. This review also systematically summarizes modern research strategies employed to investigate OBPs/CSPs functions, including high-throughput omics technologies, RNA interference, CRISPR-Cas9 gene editing, and molecular docking, while discussing the potential of targeting these proteins for developing novel insecticides and resistance management strategies. Although significant progress has been made in laboratory studies, the practical application of OBPs/CSPs-mediated resistance mechanisms still faces multiple challenges. Future research should prioritize multi-gene targeting strategies, cross-species functional validation, and field trial implementation to facilitate the development of green and precise pest control approaches based on OBPs and CSPs, thereby offering new pathways for sustainable agriculture.

## 1. Introduction

Pests pose severe threats to crop yield and quality through direct herbivory and by acting as vectors for viral diseases [1]. Although chemical control remains the primary method for pest management, the prolonged and indiscriminate use of insecticides has resulted in varying levels of resistance in pest populations [2]. Insecticide resistance represents a major challenge in contemporary pest management. Most studies on insecticide resistance have primarily focused on three mechanisms: target-site insensitivity caused by mutations, enhanced metabolic detoxification mediated by key enzymes such as P450 monooxygenases (P450s), carboxylesterases (CarEs), glutathione S-transferases (GSTs), and uridine diphosphate (UDP)-glycosyltransferases (UGTs), and penetration resistance resulting from increased synthesis of cuticular hydrocarbons [3,4,5]. Nevertheless, the intrinsic complexity of resistance mechanisms, exacerbated by the continual introduction of novel active ingredients with unprecedented modes of action, means our understanding of insecticide resistance remains incomplete. Recent studies have indicated that chemosensory-related proteins, particularly odorant-binding proteins (OBPs) and chemosensory proteins (CSPs), may play a non-negligible role in the development of insecticide resistance [6,7,8]. Traditionally, these two classes of proteins have been considered specifically involved in insect chemosensory processes, such as host location, mate recognition, and predator avoidance [9]. However, these studies suggest that OBPs and CSPs can also influence insect susceptibility to insecticides through both direct and indirect mechanisms.

OBPs and CSPs are small soluble proteins that are extensively expressed in the insect sensillar lymph. Their primary function is to bind and transport hydrophobic odorant molecules to olfactory receptors, thereby initiating neural signal transduction [10]. However, recent studies show that the altered expression levels of these proteins are significantly correlated with insecticide resistance phenotypes. For instance, an OBP located in *Anopheles gambiae* legs has been shown to bind pyrethroids, preventing the insecticide from reaching its target site and conferring resistance [11]. Similarly, a CSP in *Nilaparvata lugens* is overexpressed upon exposure to imidacloprid, this protein sequesters the insecticide and enhances metabolic detoxification, ultimately leading to resistance to imidacloprid [12]. These findings broaden our understanding of the functional repertoire of OBPs and CSPs, suggesting that they may perform atypical roles beyond chemical communication, such as serving as buffers or transport carriers in detoxification processes. At present, research on their contribution to insecticide resistance remains in its infancy. The precise molecular mechanisms, particularly their interactions with established resistance pathways, require systematic elucidation. This review summarizes recent advances in understanding how OBPs and CSPs contribute to insecticide resistance in pests. It explores the mechanisms through which pests utilize these proteins to recognize and adapt to insecticides, offering new perspectives for elucidating the molecular basis of resistance evolution. This review primarily focuses on the mechanisms of action and efficacy of insecticides developed and used in agricultural settings. Our aim is to provide a theoretical foundation for developing more scientifically grounded and effective pest management strategies.

This review conducted systematic searches in Web of Science, PubMed and CNKI databases for literature published between 1994 and 2025, using the following keyword combinations: (“odorant-binding protein” OR “chemosensory protein”) AND (“insecticide” OR “pesticide”) AND (“resistance”) AND (“insect”) OR (“function” OR “molecular characteristics” OR “detoxification” OR “technologies”) OR (“synergistic Strategies” OR “monitor”). We identified relevant studies through a three-step screening process comprising deduplication, title-abstract screening, and full-text assessment, ultimately including 82 studies that met our quality criteria.

## 2. Structural and Functional Diversity of OBPs and CSPs

### 2.1. Molecular Characteristics

OBPs are low-molecular-weight, water-soluble proteins, typically consisting of 120–150 amino acid residues [13,14], that are highly expressed in the sensillar lymph [15]. A key feature of OBPs is the presence of conserved cysteine residues that form disulfide bridges. This cross-linking stabilizes a predominantly α-helical structure that folds into a hydrophobic binding cavity. This architecture forms the conserved structural framework of OBPs and is vital for binding and solubilizing hydrophobic odorants in the aqueous sensillar lymph. OBPs are classified based on the number of conserved cysteine residues: Classical OBPs (six cysteine forming three disulfide bonds), Plus-C OBPs (more than six cysteines and a proline residue, typically forming two or three disulfide bonds), Minus-C OBPs (four or five cysteines forming two disulfide bonds), and Atypical OBPs (featuring an extended C-terminal region) [16]. The formation of these disulfide bonds is critical for maintaining structural stability and is essential for the ligand-binding capacity of OBPs [17]. CSPs are small, water-soluble proteins with a molecular weight similar to that of OBPs [18]. First identified in *Drosophila melanogaster* [19], their structure is stabilized by two disulfide bonds bridging four conserved cysteine residues [20]. The protein folds into a compact globular structure predominantly composed of α-helices. The structure is distinguished by a hydrophilic surface and a hydrophobic internal cavity, which enables the binding of lipophilic ligands [10]. The disulfide linkages in CSPs introduce fewer structural restraints compared to the rigid framework of OBPs, thereby endowing CSPs with enhanced conformational plasticity. This structural plasticity enables CSPs to recognize and bind a broader spectrum of ligands. It also underpins their remarkable stability, enabling retained physiological activity under denaturing conditions, including high temperatures and organic solvents [21].

### 2.2. Functional Diversity

Initially, OBPs and CSPs were believed to be restricted to chemosensory tissues, with the highest expression levels detected in the antennae. Their primarily function was understood to be the recognition and transport of signaling molecules that mediate essential behaviors, including host localization, mating and oviposition [22,23,24]. A pivotal advance in chemoreception occurred in 1981, when Vogt et al. identified the first pheromone-binding protein (PBP) in the antennae of *Antheraea polyphemus* [25]. This seminal discovery established OBPs as a central focus of subsequent research. Through the application of RNA interference (RNAi) and functional genomic approaches, investigators Yi et al. (2024) identified two OBPs in *Spodoptera exigua* that play critical roles in the recognition of sex pheromones [26]. Their findings demonstrate that these OBPs are key regulators of reproductive behavior in this pest species. Han et al. [27] demonstrated that CrufCSP3, a chemosensory protein expressed in the antennae of *Cotesia ruficrus*, is involved in the perception of volatiles emitted by the *Helicoverpa armigera*. The team identified four key amino acid residues essential for binding host-associated compounds through a combination of fluorescence competitive binding assays and site-directed mutagenesis, pinpointing the structural determinants of this interaction. Furthermore, RNAi experiments confirmed that CrufCSP3 plays an essential role in mediating host localization and parasitization behavior.

Nevertheless, a growing number of studies have detected OBPs and CSPs are also expressed in non-olfactory tissues, suggesting functions beyond chemosensation. Furthermore, Yu et al. [28], used Western blot to demonstrate the presence of LmigOBP2 not only in the antennae but also in the mouthparts of *Locusta migratoria*. In a separate study, CSPs were identified in the cuticle and hemolymph of *Schistocerca gregaria* [29]. Their expression in these non-sensory tissues suggests a potential functional association with developmental processes in *S. gregaria*. Jeong et al. employed CRISPR/Cas9-mediated knockout and behavioral assays demonstrated that a specific OBP expressed in the gustatory organs of *Drosophila* is involved in taste perception and mediates avoidance of harmful substances such as quinine. Their study revealed a dose-dependent dual mechanism: low-concentration quinine is sequestered by the OBP, blocking receptor interaction, whereas high concentrations saturate the OBP’s binding capacity, directly triggering avoidance behavior and feeding suppression. These complementary mechanisms allow insects to balance nutrient intake with avoidance of toxic compounds [30]. A 2024 study by Gravino et al. reported a CSP in the saliva of *Myzus persicae* that functions as an effector protein: it is introduced into the host plants during feeding to inhibit plant immunity [31]. This finding considerably broadens the functional scope of CSPs in insect-plant interactions by revealing a novel, non-sensory physiological role.

## 3. Mechanisms Linking OBPs/CSPs to Insecticide Resistance

### 3.1. Enhanced Detoxification and Metabolic Resistance

**Figure 1:**
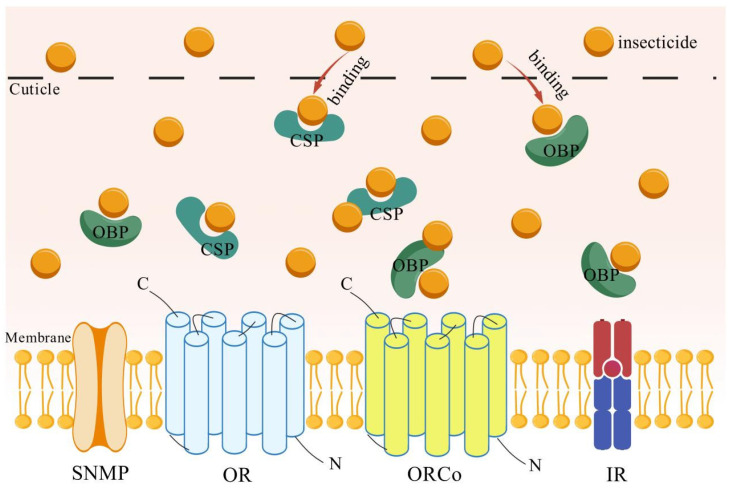
Binding of OBPs/CSPs to insecticides: sequestering insecticides away from their target sites. Abbreviations: SNMP, sensory neuron membrane protein; OR, odorant receptor; ORCo, odorant receptor co-receptor; IR, ionotropic receptor. Figure was created using BioGDP (https://biogdp.com/).

Insecticide Binding and Transport: Traditionally, OBPs and CSPs have been recognized for binding odor molecules and mediating chemosensation. However, growing evidence indicates that these olfactory-related proteins also contribute to insecticide resistance, revealing an underappreciated facet of their functional versatility. OBPs and CSPs are capable of binding exogenous insecticides in a sponge-like manner, sequestering them and preventing their toxic effects within the insect (Figure 1). However, OBPs and CSPs themselves lack the ability to metabolize these insecticides, a phenomenon referred to as “sequestration” [32]. Enhancing the binding affinity between OBPs/CSPs and exogenous insecticides constitutes an important mechanism in the development of insecticide resistance [33]. In coleoptera, upon exposure to dichlorvos for 12–72 h, *Tribolium castaneum* exhibited significant upregulation of *TcasCSP10* and *TcasOBPC01* expression [34]. Furthermore, RNAi-mediated knockdown of these proteins increased the susceptibility of *T. castaneum* to dichlorvos. These findings indicates that the enhanced binding affinity of TcasCSP10 and TcasOBPC01 for dichlorvos reduces the effective insecticide concentration at the target site, a molecular mechanism that contributes to insecticide tolerance. Through a combination of binding assays, molecular docking, and site-directed mutagenesis, Liang et al. [35] established that RhorOBP1 in *Rhaphuma horsfieldi* recognizes not only natural odorants but also the insecticide chlorpyrifos. Their results delineate a structural basis for this interaction, revealing considerable affinity between chlorpyrifos and the RhorOBP1 binding pocket. These findings provide strong evidence for the ligand-binding promiscuity of OBPs and their functional involvement in both chemosensation and insecticide recognition. In hemiptera, RNAi-mediated knockdown of *NlugOBP5* expression in an insecticide-resistant strain of *Nilaparvata lugens* significantly elevated its susceptibility to chlorpyrifos. The subsequent insecticide treatment caused a marked increase in mortality [36], demonstrating that NlugOBP5 is a key gene conferring resistance to chlorpyrifos in *N. lugens*. Studies in lepidoptera have demonstrated that the coordinate expression of CSPs and metabolic cytochrome P450 enzymes in *Bombyx mori* following abamectin exposure, indicating a potential functional coordination between CSPs and detoxification pathways in insecticide resistance [37]. Additionally, evidence of OBPs/CSPs binding insecticides and reducing their toxicity has been reported in hymenoptera [38].

Regulation of Detoxification Pathways: While cytochrome P450 monooxygenases have long been considered a principal mechanism of insecticide resistance [39], recent studies suggest a more complex picture. Furthermore, in *Plutella xylostella*, exposure to high concentrations of permethrin resulted in the upregulation of the CYP4M14, CYP305B1 and *PxylCSP8* [40], transcriptomic findings suggest a connection between detoxification mechanisms and chemosensation in the diamondback moth, but the study did not delve into the specific mechanisms of action. During an investigation into the insecticidal mechanism of eugenol, Zhang et al. [41] observed that eugenol significantly inhibited neural carboxylesterases and detoxification glutathione S-transferases (GSTs) in *T. castaneum*, ultimately leading to paralysis or death. Their study further revealed that multiple gene families, including CYPs, UGTs, GSTs, OBPs, CSPs, and ABC transporters, are involved in the stress response of *T. castaneum* to eugenol. Subsequent qRT-PCR analysis and RNAi experiments demonstrated that silencing either *TcOBPC11* or *TcGSTs7* significantly increased mortality in eugenol-treated groups, providing direct evidence that both *OBP* play essential roles in the metabolic detoxification of eugenol in *T. castaneum*. Chen et al. [42] found that RNAi silencing of *TcGSTd1* in *T. castaneum* significantly downregulation of *OBPs* and *CSPs*, indicating a coordinated regulatory relationship between olfactory proteins and GSTs in the detoxification process. These results demonstrate that CSPs and OBPs modulate GST-mediated detoxification pathways, offering novel insights into how olfactory proteins contribute to insecticide resistance.

### 3.2. Behavioral Avoidance

In pest species, OBPs and CSPs recognize harmful substances such as insecticides, enabling them to avoid hazardous hosts and evade toxic crops. DhelOBP4 and DhelOBP21 from *Dastarcus helophoroides* demonstrate high binding affinity for and effectively recognize the pesticides cyetpyrafen, chlorfenapyr, and spirodiclofen. Furthermore, RNAi targeting *DhelOBP4* and *DhelOBP21* significantly attenuated the repellent effects of these three pesticides on *D. helophoroides*, confirming that DhelOBP4 and DhelOBP21 serve as key molecular targets involved in the olfactory recognition of cyetpyrafen, chlorfenapyr, and spirodiclofen in this species [43]. Certain OBPs in *Drosophila* trigger avoidance behavior toward aversive compounds such as quinine, leading to the cessation of feeding [30]. These results support a model in which OBPs contribute to evasion-based resistance against toxins by modulating repellent behaviors. On the other hand, insects can also mitigate toxic effects through adaptive metabolic and behavioral shifts. For instance, in *Apis mellifera*, OBPs bind to the neonicotinoid insecticide cycloxaprid (CYC), which reduces overall food consumption yet does not prevent the ingestion of the toxin itself [44]. This feeding behavior significantly enhances the survival and reproductive capacity of *A. mellifera*. Studies have also shown that upon reaching sublethal internal concentrations of abamectin from ingested treated food, *B. mori* exhibits a marked increase in CSP expression, resulting in the termination of feeding [37]. These findings indicate that OBPs and CSPs mediate adaptive behaviors in response to chemical threats, substantially reducing insecticide-induced harm.

## 4. Methodological Advances in Studying the Roles of OBPs and CSPs in Insecticide Resistance

### 4.1. Omics Technologies

As early as 2009, RNA sequencing (RNA-Seq) was recognized as a cornerstone of transcriptomics, and its advantages have since underpinned diverse applications, including insecticide-resistance research [45]. Following this, 454 pyrosequencing technology was successfully applied to profile the transcriptome of *Pararge aegeria*, demonstrating the significant utility of RNA-Seq for non-model organisms whose genomic sequences had not yet been determined [46]. In 2010, Marutani-Hert et al. [47] performed transcriptomic analysis on detoxification-related genes (including CYPs and GSTs) in *Diaphorina citri* after treatment with a sublethal dose of the insecticide imidacloprid. This study provided a foundational dataset and methodological framework for subsequent identification of resistance genes through comparative analysis of OBPs and CSPs expression levels between insecticide-resistant and susceptible strains. Subsequently, Liu et al. [48] identified BtabCSP1 as a key gene conferring resistance in *Bemisia tabaci* by comparing two distinct strains using Sanger sequencing. This study represents one of the most direct demonstrations of applying transcriptomic analysis to identify insecticide resistance genes. Proteomics has emerged as another rapidly advancing field of research and has been increasingly utilized in recent years to investigate mechanisms underlying insect development and insecticide resistance [49]. Hassan et al. [50] employed one-dimensional electrophoresis (1-DE) and two-dimensional gel electrophoresis (2-DE) to identify an optimized protein extraction method for larval and adult tissues of *P. xylostella*, specifically the TCA/acetone precipitation protocol supplemented with 40 mM dithiothreitol (DTT). This method provided crucial technical support for subsequent functional studies on the roles of OBPs and CSPs in insecticide resistance in lepidoptera. Following the treatment of *P. xylostella* with gelomycin III, proteins from treated and control groups were separated by 2-DE and compared, initial identifying of 31 differentially expressed proteins. Subsequent molecular biological assays ultimately pinpointed PxylCSP2 as the target through which gelomycin III affects oviposition behavior in *P. xylostella* [51]. This demonstrates the advantage of proteomics over transcriptomics for addressing functional biological questions.

### 4.2. Functional Validation

RNAi is widely employed as a powerful tool for functional genomics validation due to its high efficiency and systematic applicability [52,53]. A common approach to validate gene function in insects involves delivering double-stranded RNA (dsRNA) through feeding or injection [54], which leads to the silencing of the target gene and subsequent loss of function. The CRISPR-Cas gene editing enables precise modification of insect DNA, its applications extend beyond triggering gene drives to include targeted editing of resistance genes, thereby overcoming insecticide resistance and offering new strategies for pest control research [55]. Molecular docking, a structure-based computational approach, predicts the spatial binding site and binding affinity between a ligand and its target [56]. This method is widely used to predict the binding capacity of OBPs and CSPs to insecticide molecules, offering robust validation that their corresponding genes are key factors in the development of insecticide resistance. To identify key genes involved in resistance to imidacloprid and cypermethrin in *Rhopalosiphum padi*, researchers employed molecular docking to screen five CSPs based on their predicted binding capacity to these insecticides. This in silico prediction was further validated through RNA interference via dsRNA injection, which confirmed that RpadCSP4 and RpadCSP6 are critical for resistance to both imidacloprid and cypermethrin [57]. Following the knockout of *BdorOBP28a-2* in *Bactrocera dorsalis* using CRISPR/Cas9 technology, subsequent treatment with malathion resulted in a significant increase in mortality, demonstrating that the BdorOBP28a-2 is critical for resistance [58]. Liu et al. [59] employed fluorescent competitive binding assays to investigate the binding capabilities of two OBPs from *Spodoptera frugiperda* to various insecticides. They complemented these experiments with molecular docking simulations to calculate the binding affinities between the OBPs and different insecticides. This combined approach provided critical insights for subsequent research into the mechanisms by which OBPs in *Spodoptera* species confer resistance to insecticides.

### 4.3. Implications for Pest Management

With the increasing prevalence of insecticide resistance in pests, the efficacy of control and monitoring strategies based on a single tactical approach has been significantly compromised, highlighting the urgent need to develop multi-targeted and sustainable pest management systems [60]. The growing understanding of the roles played by OBPs and CSPs in insect resistance mechanisms offers novel perspectives and approaches for controlling harmful insect pests [34]. Based on the structures of OBPs and CSPs, high-throughput screening and computer-aided design can be utilized to develop small-molecule inhibitors or competitive ligands that disrupt their binding activity [61]. This approach breaks away from the long-standing singular focus on neural targets and metabolic detoxification, thereby diversifying the approaches available for pest control by targeting sensory perception pathways.

### 4.4. New Targets for Insecticide Design

Based on the three-dimensional structures of OBPs and CSPs, particularly the characteristics of their ligand-binding domains [62], insecticide molecules are recognized and bound by these proteins. This interaction results in the sequestration or hijacking of insecticide molecules, preventing them from reaching their target sites (e.g., neural receptors or enzymes), thereby reducing toxicity and enhancing insect tolerance to the insecticides. Consequently, disrupting the interaction between OBPs/CSPs and insecticides has emerged as a novel strategy for addressing insecticide resistance. The core of this approach lies in employing structural biology methods—such as molecular docking and computer-aided design—to develop small-molecule inhibitors or mimetic ligands that competitively occupy the binding pockets of OBPs/CSPs. This intervention aims to disrupt the “molecular sponge”-like detoxification mechanism and restore the efficacy of conventional insecticides [63]. The crystal structure of the PBP from *H. armigera* has been resolved. Key residues critical for ligand recognition, Lys94 and Lys138, were identified, providing further insight into the mechanism of ligand binding and release by PBPs [64]. Another critical aspect influencing binding characteristics is binding affinity. Gao et al. [65] predicted the three-dimensional structure of RpCSP6 from *R. padi* using AlphaFold2, and performed molecular docking simulations along with binding energy calculations between RpCSP6 and deltamethrin using AutoDock 4. The binding affinity was further validated through fluorescent competitive binding assays, demonstrating the reliability of computational simulations in studying such interactions. These findings strongly support the feasibility of designing small molecules that interact with the ligand-binding domain of OBPs/CSPs by leveraging three-dimensional structural insights and computational predictions. Such strategies aim to develop inhibitors that disrupt OBP/CSP-insecticide interactions through two primary mechanisms: the competitive binding of small molecules to the protein domain to compete with insecticide for the same binding site, or the induction of protein denaturation. This approach provides a rational strategy for countering insecticide resistance.

### 4.5. Resistance Monitoring

The conventional approach to resistance monitoring is based on bioassays, wherein insects are exposed to a range of insecticide concentrations, and the dose-mortality response is analyzed to generate a toxicity regression line [32]. This monitoring method is only applicable when pests have already developed high-level resistance. In contrast, using OBPs/CSPs expression profiles as biomarkers for early detection enables identification at the initial stages of resistance development, significantly shortening the response time. Resistance monitoring in North China revealed that field populations of *Aphis gossypii* developed resistance to five common insecticides by 2022 [66]. Notably, a prior study in 2021 had already revealed that AgosCSP5 expression in this aphid is upregulated by multiple pesticides [67]. Similarly, vial bioassays conducted in 2019 on regional populations of *B. tabaci* indicated high resistance to thiamethoxam—a phenomenon consistent with the 2016 finding that this neonicotinoid significantly upregulates BtabCSP1 expression in the whitefly [48,68]. An analogous pattern of RpadCSP induction under insecticide exposure has also been reported in *R. padi*, suggesting a conserved transcriptional response across pest species [57,69].

### 4.6. Synergistic Strategies

The combined application of conventional insecticides and inhibitors targeting OBPs/CSPs represents a viable approach for overcoming protein-mediated insecticide resistance, thereby improving overall pest control outcomes. Furthermore, RNAi technology has emerged as a robust tool for gene functional analysis and has proven highly effective in silencing OBPs and CSPs [54]. The injection of dsRNA into insects achieves high interference efficiency, making the combination of RNAi-based agents with conventional insecticides a viable strategy [70]. DsRNA can be delivered into insects through several methods, including injection, soaking, and feeding. A comprehensive review has concluded that feeding-based RNAi delivery achieves high interference efficiency in the vast majority of coleopteran insects [71], although notable exceptions exist in certain insect species such as the *L. migratoria* [72], where this method demonstrates limited effectiveness. To enhance RNAi efficiency, numerous novel technologies have been integrated with RNAi in recent years. For example, cationic liposomes have been successfully used to deliver dsRNA into insects, as notably demonstrated in *Drosophila suzukii* [73]. Another common technique for enhancing RNAi efficiency is nanoparticle-mediated delivery. By encapsulating dsRNA, nanoparticles protect it from degradation and significantly improve its cellular uptake and gene silencing efficiency in insects [74]. The rapid advancement of RNAi technology provides a strong foundation for developing OBPs/CSPs inhibitors. The combination of traditional insecticides with OBPs/CSPs inhibitors leverages their synergistic effects, offering a novel and promising strategy for improving pest control.

## 5. Challenges and Future Directions

### 5.1. Functional Redundancy

The overlap in ligand binding specificity among different OBPs/CSPs implies that silencing a single gene may not often yield significant phenotypic changes, as insects can compensate for the lost function through other proteins. This functional redundancy thus poses a major challenge for overcoming insecticide resistance via single-gene targeting strategies. In the parasitoid wasp *Cotesia vestalis*, three OBPs have been identified which are organized in a cluster and exhibit high expression levels. Studies demonstrate that concurrent RNAi-mediated silencing of these three OBP genes results in a more substantial inhibition of host-seeking behavior compared with targeting individual genes [75]. This result provides direct evidence for functional redundancy among OBPs.

In *Apis cerana*, exposure to the neonicotinoid insecticide imidacloprid induces the significantly upregulates the expression of *AcerOBP10* in the venom gland [76]. Moreover, the expression of *AcerOBP17*, which is predominantly expressed in the legs, is also elevated under imidacloprid exposure. Critically, fluorescence competitive binding assays have demonstrated that *AcerOBP17* can bind to imidacloprid, suggesting that its upregulation confers a protective effect by reducing the insecticide’s toxicity [77]. These findings illustrate that targeted intervention against a single protein is often insufficient to completely overcome insecticide resistance, as functional redundancy among other proteins can compensate for the loss. Consequently, effective pest control typically requires simultaneous targeting of multiple proteins, which significantly increases the complexity of intervention strategies.

### 5.2. Cross-Species Variability

In the cotton aphid (*Aphis gossypii*), the CSP family exhibits a unique pattern of “conservation and loss coexistence”: AgosCSP2 and AgosCSP3 are completely absent in some field populations, while AgosCSP5 has been consistently identified in multiple studies as a key gene mediating imidacloprid resistance, with its expression level significantly positively correlated with resistance [67]. However, this mechanism is not universally applicable across species. For example, in the Chinese honey bee (*Apis cerana*), exposure to the same insecticide involves a shift in key resistance genes to members such as AcerOBP17 [77]. This indicates that the resistance network mediated by CSPs/OBPs is highly species-specific (Table 1): molecules that play critical roles in one pest species often cannot be directly applied to another. Moving forward, systematic research on target species is necessary, including genome-wide family identification, spatiotemporal expression profiling, and thermodynamic characterization of insecticide–protein interactions, to provide a replicable framework for the precise development of species-specific interference strategies.

### 5.3. Field Applicability

Even if key genes conferring insecticide resistance are identified in the laboratory and corresponding countermeasures are developed, translating these strategies into practical field applications remains profoundly challenging. The complexity of field environmental conditions can significantly compromise the efficacy of approaches that proved successful under controlled laboratory settings. For example, when investigating the binding properties of OBPs and CSPs to ligand molecules using fluorescence competitive binding assays, studies have revealed that the binding affinity of the same protein-ligand pair varies under different temperature conditions. Furthermore, the emission wavelength in fluorescence quenching assays shifts in response to changes in both temperature and solvent composition [78]. Moreover, under field conditions, temperatures fluctuate constantly, which can lead to continuous alterations in the binding properties of insect proteins. Additionally, insects maintain symbiotic relationships with environmental microorganisms. Through evolutionary and ecological processes, these interactions give rise to highly specialized, host-specific microbial communities within their guts [79]. Recent studies have revealed that insect gut microbiota play a crucial role in host survival by facilitating detoxification processes and enhancing innate resistance to insecticides [80]. These findings provides both theoretical and experimental support for the hypothesis that microbial communities colonizing insects may degrade or modify OBPs/CSPs-targeting interfering agents, thereby reducing their efficacy. Therefore, any promising laboratory discovery must be rigorously validated and optimized through field trials under real-world conditions to ensure its stability, efficacy, and economic viability.

**Table 1:** Insect OBPs/CSPs associated with insecticides.

Species	Order	CSPs/OBPs	Developmental Stage	Insecticide	Experimental Evidence	Reference
*Tribolium castaneum*	Coleoptera	CSP10 and OBPC01	Larvae	Dichlorvos	Upregulated; increased susceptibility after RNAi	[34]
*Rhaphuma horsfieldi*	Coleoptera	OBP1	Adult	Chlorpyrifos	High binding affinity	[35]
*Dastarcus helophoroides*	Coleoptera	OBP4 and OBP21	Adult	Cyetpyrafen, chlorfenapyr, spirodiclofen	High binding affinity; disappearance of avoidance behavior after RNAi	[43]
*Ceutorhynchus asper*	Coleoptera	OBP12	Female	λ-Cyhalothrin	High binding affinity	[81]
*Anopheles gambiae*	Diptera	SAP2	Adult	Pyrethroid	Upregulated; high binding affinity; increased susceptibility after RNAi	[11]
*Bactrocera dorsalis*	Diptera	OBP28a-2	Adult	Malathion	Upregulated; high binding affinity; increased susceptibility after CRISPR/Cas9 mutagenesis	[58]
*Aphis gossypii*	Hemiptera	CSP1, CSP4 and CSP5	Adult	Cyantraniliprole	Upregulated; ctopic expression confers retained insecticide resistance; increased susceptibility after RNAi	[6]
*Rhopalosiphum padi*	Hemiptera	CSP4 and CSP5	Adult	Thiamethoxam	Upregulated; high binding affinity; increased susceptibility after RNAi	[7]
*A. gossypii*	Hemiptera	CSP1 and CSP4	Adult	Thiamethoxam	Upregulated; high binding affinity; ctopic expression confers retained insecticide resistance; increased susceptibility after RNAi	[8]
*Nilaparvata lugens*	Hemiptera	CSP2, CSP4, CSP5, CSP7, CSP12 and CSP15	Adult	Imidacloprid	Upregulated; increased susceptibility after RNAi	[12]
*N. l* *ugens*	Hemiptera	OBP5	Adult	Chlorpyrifos	Upregulated; high binding affinity; increased susceptibility after RNAi	[36]
*N. lugens*	Hemiptera	OBP3	Larvae	Nitenpyram, sulfoxaflor	Upregulated; increased susceptibility after RNAi	[82]
*Bemisia tabaci*	Hemiptera	CSP1	Adult	Thiamethoxam	Upregulated	[48]
*R. padi*	Hemiptera	CSP4, CSP5, CSP6 and CSP10	Adult	Imidacloprid	High binding affinity; increased susceptibility after RNAi	[57]
*R. padi*	Hemiptera	CSP4 and CSP6	Adult	Beta-cypermethrin	High binding affinity; increased susceptibility after RNAi	[57]
*R. padi*	Hemiptera	CSP6	Larvae and adult	Deltamethrin	Upregulated; increased susceptibility after RNAi	[65]
*Apis cerana*	Hemiptera	OBP17	Adult	Imidacloprid	Upregulated; high binding affinity; Enhanced the electrophysiological response after RNAi	[77]
*Meteorus pulchricornis*	Hymenoptera	OBP6	Adult	Phoxim, chlorpyrifos, chlorfenapyr	High binding affinity	[38]
*Plutella xylostella*	Lepidoptera	CSP2	Adult	Rhodojaponin III	Upregulated; reduced oviposition after RNAi	[51]
*Spodoptera frugiperda*	Lepidoptera	OBP18	Larvae	Spinetoram, chlorfenapyr, chlorpyrifos, indoxarweb	Upregulated; increased susceptibility after RNAi	[83]

## 6. Conclusions

With the growing challenge of global agricultural pest resistance to chemical insecticides, OBPs and CSPs have attracted considerable research interest as key players in the development of insecticide resistance. These proteins play a central role in insect chemoreception, behavioral regulation, and physiological metabolism. They contribute to insecticide resistance not only indirectly by influencing detoxification processes but also directly through modulating pest behavior and interactions with target sites. OBPs and CSPs contribute to insecticide resistance through multiple mechanisms. In detoxification metabolism, these proteins function by sequestering insecticides and altering their transport/bioavailability, thereby modulating detox enzyme activity. For instance, the upregulation of specific CSPs in *B. mori* enhances metabolic detoxification capabilities against abamectin insecticides [37]. In behavioral adaptation, these proteins mediate chemosensory recognition that enables pests to detect and avoid toxic environments. In the beetle *D. helophoroides*, both DhelOBP4 and DhelOBP21 exhibit high binding affinity to the three pesticides. RNAi-mediated knockdown of these OBPs disrupted olfactory-guided behavior and abolished the avoidance response to these pesticides, demonstrating their critical role in facilitating active evasion of insecticidal toxicity [43]. Breakthroughs in molecular biology have provided novel perspectives for understanding resistance mechanisms. X-ray crystallography has elucidated the precise binding modes between proteins and ligands [64]. This finding provides a solid theoretical foundation for structure-guided insecticide design and, together with emerging technologies, thereby paves the way for novel resistance-management strategies. RNAi-mediated silencing of OBP or CSP genes significantly impairs both chemosensory perception and insecticide tolerance in targeted pest populations. Meanwhile, delivery systems based on nanocarriers and engineered bacteria have markedly improved the field stability and efficacy of RNAi formulations [74]. Artificial intelligence is transforming the paradigm of pesticide discovery: machine learning algorithms can now prospectively identify small-molecule inhibitors with high predicted binding affinity for OBPs/CSPs, whereas molecular dynamics simulations provide a dynamic, atomistic perspective for rational insecticide design [65]. Future research should prioritize functional validation across a broad phylogenetic spectrum of pest species, particularly those that pose imminent threats to global food security. CRISPR/Cas9-mediated germline knockouts, combined with rigorously controlled backcrossing, enable unequivocal quantification of the contribution of individual OBP or CSP genes to the development and extent of insecticide resistance. Concurrently, well-replicated, multi-site field trials should be conducted under real-world agricultural conditions to rigorously evaluate the efficacy, durability, and environmental safety of resistance-management interventions. Establishing a global resistance-monitoring network coupled with an open-access, cloud-based database will enable the real-time surveillance of the spatiotemporal distribution and evolutionary dynamics of resistance-associated alleles across pest metapopulations.

In summary, elucidating the mechanisms by which OBPs and CSPs mediate insecticide resistance provides unprecedented opportunities for developing sustainable pest-management strategies. By integrating molecular insights with emerging technologies, we can now design targeted resistance-management approaches that are highly effective, precise, and environmentally sustainable. Such advances will help safeguard global food security and accelerate the transition toward sustainable agricultural systems.

## Data Availability

No new data were created or analyzed in this study. Data sharing is not applicable to this article.
